# The Dimensional Obsessive-Compulsive Scale: Development and Validation of a Short Form (DOCS-SF)

**DOI:** 10.3389/fpsyg.2017.01503

**Published:** 2017-09-05

**Authors:** Thomas Eilertsen, Bjarne Hansen, Gerd Kvale, Jonathan S. Abramowitz, Silje E. H. Holm, Stian Solem

**Affiliations:** ^1^OCD-Team, Haukeland University Hospital Bergen, Norway; ^2^Department of Clinical Psychology, University of Bergen Bergen, Norway; ^3^Department of Psychology and Neuroscience, University of North Carolina at Chapel Hill Chapel Hill, NC, United States; ^4^Department of Psychology, Norwegian University of Science and Technology Trondheim, Norway

**Keywords:** psychometric properties, obsessive-compulsive disorder, brief questionnaire, evidence-based assessment, dimensional obsessive-compulsive scale short-form

## Abstract

Accurately and reliably measuring the presence and severity of Obsessive-Compulsive Disorder (OCD) symptoms is essential for both routine clinical work and research. The current study investigated psychometric properties of the dimensional obsessive-compulsive scale-short form (DOCS-SF). DOCS-SF was developed and validated in Norwegian. DOCS-SF contains a checklist with four symptom categories and five severity items scored on a zero to eight scale yielding a total score of 0–40. Data were collected from adults with a current diagnosis of OCD (*n* = 204) and a community comparison group (*n* = 211). The results provided evidence of internal consistency and convergent validity, although evidence for discriminant validity was mixed. Evidence was also found for diagnostic sensitivity and specificity, and treatment sensitivity. The analyses suggested a cut-off score of 16. In summary, the data obtained proved similar to studies published on the original dimensional obsessive-compulsive scale. There is strong evidence for the reliability and validity of the DOCS-SF for assessing OCD symptoms in individuals with this condition and in non-clinical individuals.

## Introduction

Obsessive-compulsive disorder (OCD) is a heterogeneous condition, and factor-analytic research has consistently found clusters of symptoms along 3–5 dimensions (Mataix-Cols et al., 2005, 2016). As a consequence of the need for a self-report measure that incorporates different symptom dimensions, Abramowitz et al. (2010) developed the 20-item Dimensional Obsessive-Compulsive Scale (DOCS).The DOCS assesses the severity of the four most consistently replicated O-C symptom dimensions: (a) contamination/washing, (b) harm obsessions/checking compulsions, (c) symmetry/ordering, and (d) unacceptable thoughts. Hoarding, which is no longer considered a presentation of OCD, is not assessed. The DOCS provides an index of severity that is independent of the number and types of obsessions and compulsions present, and consequently does not confound the number of different types of obsessions and compulsions with severity. Rather, the DOCS measures symptom severity as a function of empirically supported parameters for each of the four dimensions: (a) time occupied by obsessions and compulsions, (b) avoidance, (c) associated distress, (d) interference with function, and (e) refraining from compulsions. Each item is rated on a 0–4 scale, yielding a total score from 0 to 80 as well as individual subscale scores ranging from 0 to 20.

Numerous studies have been conducted evaluating the psychometric properties of the DOCS in clinical and non-clinical samples and in various languages (Abramowitz et al., 2010; Enander et al., 2012; Wang et al., 2012; Kim et al., 2013; Ólafsson et al., 2013; López-Solà et al., 2014; Melli et al., 2015). The mean total scores among OCD samples have ranged from 25.3 to 32.7 with standard deviations ranging from 14.0 to 19.8. For students/comparison group the mean scores have ranged from 10.8 to 13.1 with standard deviations ranging from 8.8 to 10.2. Cronbach's alpha values have ranged from 0.87 to 0.95 for the total DOCS score. Two studies have examined test-retest reliability (Abramowitz et al., 2010; López-Solà et al., 2014), finding *r*s of 0.66 and 0.81 for OCD samples, and 0.43 for a student sample. Correlations with the Yale-Brown Obsessive-Compulsive Scale (Y-BOCS; Goodman et al., 1989) interview, which provides a global measure of severity, ranged from 0.47 to 0.64, and correlations with the self-report Obsessive-Compulsive Inventory-Revised (OCI-R; Foa et al., 2002) ranged from 0.69 to 0.86. The DOCS also appears sensitive to treatment effects, and Abramowitz et al. (2010) found that a cut-off score of 21 optimally differentiated between individuals diagnosed with OCD and those with other anxiety disorders with 70% sensitivity and 70% specificity; and a cut-off of 18 optimally differentiated OCD patients from comparison group with 78% sensitivity and 78% specificity. López-Solà et al. (2014) found a cut-off of 15 being optimal, giving 71% sensitivity and 76% specificity against non-clinical comparison group. Taken together, these findings provide strong evidence for the DOCS as a reliable and valid measure of the severity of obsessive-compulsive symptoms in clinical and non-clinical samples.

Although, the DOCS represents an improvement over previously developed self-report measures, clinicians and researchers are constantly seeking more efficient measures that still provide a reliable and valid measurement of the presence and severity of OCD symptoms. Accordingly, we undertook the present study to develop the Dimensional Obsessive Compulsive Scale-Short Form (DOCS-SF) and to evaluate its psychometric characteristics.

The DOCS-SF is a 5-item self-report questionnaire developed on the basis of and inspired by the original DOCS, as well as Y-BOCS. DOCS-SF was developed and validated in Norwegian. So far no English version of DOCS-SF has been developed and tested. Lifetime prevalence of OCD in Norway is 1.6%, and higher for women than men (Kringlen et al., 2001). A major difference between DOCS-SF and the original DOCS is that the former uses a checklist replacing the different sub-scales of the DOCS. Respondents indicate on a checklist if they experience any of the unpleasant and intrusive thoughts (obsessions) that are characteristic of the four symptom dimensions assessed by the original DOCS. There is a definition for each of the symptom dimension listed on the questionnaires. This is different from the multiple examples for each dimension in the original DOCS. A fifth option “other intrusive and unpleasant thoughts” is also available. Here, respondents can specify any obsessive-compulsive symptoms they experience that do not fit with the four symptom dimensions. A benefit of this is shorter administration time. As a result DOCS-SF might be used as a brief screening tool and for session-to-session ratings. On the other hand, the DOCS-SF does not provide estimates of symptom scores for the various obsessive-compulsive symptom dimensions. This could be confusing to individuals and might lead to less exact or valid responses. This is particularly relevant for the “other” category of the DOCS-SF checklist. For example, a patient might include depressive or other anxiety symptoms within this category, and consider these non-OCD symptoms when responding to the five severity items. In the second part of the DOCS-SF, respondents rate the severity (during the past week) of the obsessions and compulsions they endorsed on the checklist. Severity is rated along the five parameters similar to those used in the original DOCS. This as evidence of the reliability and validity of these parameters has been previously reported. The DOCS-SF items, however, are rated from 0 to 8 (as opposed to 0–4 on the original DOCS). The change from 0–4 to 0–8 Likert scale was done in order to yield a range of severity sum scores similar to the range found in the Y-BOCS.

If its psychometric properties are sound, the DOCS-SF might provide general practitioners' (GP) with a brief initial screening for OCD symptoms before GPs refer them to specialist assessment and treatment, improve planning and evaluation of treatment effects among specialist health care providers, and give researchers a new valid and time-efficient measure of OCD symptom severity. We hypothesized that the DOCS-SF would show evidence of good internal consistency and convergence with other measures of OCD symptoms (i.e., the Y-BOCS interview, Y-BOCS self-report, and OCI-R). We also predicted that we would find evidence of discriminant validity as evident by weaker correlations with measures of depression and anxiety. We further expected that the DOCS-SF would be sensitive to treatment and that scores on the DOCS-SF would be higher for patients with OCD than for non-clinical comparison group. Finally, we sought to provide cut-off scores with optimal sensitivity and specificity for distinguishing patients with OCD from non-clinical comparison group.

## Methods

### Participants and procedure

The total sample included 415 adults in two groups: 204 patients diagnosed with OCD, and 211 unselected adults (the comparison group). The study selected and used data from a medical quality registry for the patient sample. Eligible patients were as follows: Patients must first be referred from their GP to specialist health care in Norway. If the specialist health care suspects OCD, they then refer the patients to an outpatient OCD clinic. All referred patients are offered to take part in a standard quality assurance procedure. The procedure includes patients filling out several questionnaires prior to their assessment session. The assessment session consist of a structured diagnostic assessment using MINI International Neuropsychiatric Interview (Sheehan et al., 1997, 1998) and the Y-BOCS interview (by trained clinical psychologists or psychiatrists). Any patients who underwent the assessment and completed the Patient Health Questionnaire (PHQ-9; Spitzer et al., 1999), Generalized Anxiety Disorder questionnaire (GAD-7; Spitzer et al., 2006), Obsessive Compulsive Inventory-Revised (OCI-R; Foa et al., 2002), DOCS-SF, and the Client Socio-Demographic and Service Receipt Inventory (CSSRI; Chisholm et al., 2000) (all measures are described further below) were used for this sample. All patients without a primary diagnosis of OCD were not drawn for the current sample, but otherwise no exclusion criteria were used. Table 1 summarizes the demographic characteristics of both groups. The average age of OCD patients was 31.0 years (*SD* = 10.5). About two-thirds (70.6%) of the patients were females, and about one-third (37.3%) of the patients presented with a comorbid disorder. The most prevalent comorbidity was depression (16.7%) and generalized anxiety disorder (10.3%). The medical quality registry was approved by the local Data Protection Official for Research (Personvernombudet). Full ethical review and approval was not required in accordance with the national and institutional guidelines.

**Table 1:** Demographic Characteristics for the OCD and comparison group.

	**Group**	
	**OCD**	**Comparison group**
Sex, % females	70.6%	71.6%
**AGE**		
*M* (*SD)*	31.0 (10.5)	31.2 (11.5)
Range	18–69	18–74
HIGHEST ATTAINED EDUCATION^*^		
Elementary school	8.9%	
High school/Vocational high school	48.2%	
3-Year college degree or more	42.9%	
**WORK STATUS**		
Working	37.6%	44.5%
Students	29.2%	46.4%
Disability benefits/work assessment allowance	17.4%	3.3%
Other forms of income	15.7%	5.7%
Marital status (% unmarried)	54.4%	47.1%
Comorbid disorders^**^	37.3%	
Depression	16.7%	
Generalized anxiety disorder	10.3%	

* *The comparison group did not report highest attained education and was not assessed for psychiatric diagnoses*.

** *The two most common comorbid diagnoses was depression and generalized anxiety disorder*.

The comparison group (age *M* = 31.2 ± 11.5, 71.6% females) was recruited through social media and mailing lists for students, asking volunteers to complete an online survey containing the following: Demographic questionnaire, DOCS-SF, OCI-R, GAD-7, PHQ-9, and finally Y-BOCS-SR. Use of online collection methods conserves resources and previous research has found online methods to have equivalent psychometric properties as paper-and-pencil methods (Holländare et al., 2010). Participants received no compensation for completing the survey. There were no significant differences in age, *t*_(413)_ = 0.16, *p* = 0.26, and sex [$X_{\left(1\right)}^{2}$ = 0.48, *p* = 0.83], between OCD patients and comparison group. A smaller proportion of the comparison group was single compared to the OCD patients [$X_{\left(1\right)}^{2}$ = 5.41, *p* = 0.20]. More patients than comparison group participants were on disability benefits/work assessment allowance or other forms of income [$X_{\left(3\right)}^{2}$ = 37.66, *p* < 0.001]. Also, applying the cut-off criteria for Y-BOCS 93% of the comparison group were classified correctly as non-clinical.

A subsample of 88 OCD participants received treatment consisting of exposure and response prevention (EX/RP) either in a concentrated 4-day format (Concentrated Exposure Treatment; Havnen et al., 2013) or in an ordinary outpatient setting with weekly sessions. The concentrated treatment mixed individual exposure therapy and group therapy and the patient to therapist ratio was 1:1. Day 1 (4 h) included psychoeducation and exposure planning; days 2 and 3 (~8 h each) were used for therapist assisted exposure; during day 4 the focus was on lessons learned and planning self-exposures for the next 3 weeks. Three months after completing treatment patients were offered an individual follow-up session. An independent assessor not otherwise involved in the patient's treatment conducted Y-BOCS interviews post-treatment. The individual treatment followed the same principles of exposure-based treatment delivered over 90 min sessions, with 16 sessions per patient. Clinical psychologists or psychiatrists with training in the use of exposure treatment delivered the treatment. A majority (80.5%) of the subsample completed the 4-day format.

### Measures

The following questionnaires were used in the study.

#### DOCS-SF

As described previously, the DOCS-SF is a brief (5-item) self-report measure derived from the original 20-item DOCS. Total scores range from 0 to 40. A test of readability was applied (Using the LIX formula). This gave a readability index score of 41 corresponding to average readability (comparable to a regular newspaper text). The type/token ratio was 51.5%, the word variation index was 48, and the word variation ratio was 87.8%. See Appendix A1 in Supplementary Material for a copy of the questionnaire.

#### Yale-brown obsessive-compulsive scale (Y-BOCS; Goodman et al., 1989)

The Y-BOCS is the most widely used tool for assessing the global severity of OCD. It contains two parts: a checklist of over 50 types of obsessions and compulsions, and a 10-item severity scale on which the most prominent obsessions and compulsions are rated. Items on the severity scale (rated from 0 to 4) assess the following parameters of obsessions (items 1–5) and compulsions (items 6–10): time, interference, distress, resistance, and control. Total scores range from 0 to 40. There is good evidence for the Y-BOCS's inter-rater reliability, test-retest reliability, and moderate to strong evidence of internal consistency (Goodman et al., 1989). A validation study of the Norwegian Y-BOCS showed evidence of good reliability and validity (Eilertsen et al., Unpublished manuscript).

The OCD group was administered the Y-BOCS by a trained clinician whereas the comparison group completed the self-report version (Y-BOCS-SR), which has shown to have good reliability and convergent validity (Grabill et al., 2008). One study has reported a correlation between Y-BOCS-SR and Y-BOCS total score of 0.79 in a clinical sample (Steketee et al., 1996). Cronbach's alpha was 0.92.

#### Obsessive-compulsive inventory-revised (OCI-R; Foa et al., 2002)

The OCI-R is an 18-item self-report tool measuring six different symptom dimensions of OCD: Washing, obsessing, hoarding, checking, ordering, and neutralizing symptoms. Participants rate the symptoms on a scale from 0 (“Not at all”) to 3 (“Extremely”). Total scores range from 0 to 72. Evidence supports its good psychometric properties, both in the original (Foa et al., 2002), and in the translated Norwegian version (Solem et al., 2010). In the current sample, the OCI-R showed adequate psychometric properties with a Cronbach's alpha of 0.83.

#### Patient health questionnaire, 9-items (PHQ-9; Spitzer et al., 1999)

The PHQ-9 is a 9-item self-report tool, based on the nine criteria for diagnosing depression in DSM-IV. The patient scores the frequency of symptoms on a scale from zero (“Not at all”) to three (“Nearly every day”). Total scores range from 0 to 27. Evidence supports the PHQ-9 as a valid instrument for measuring depression with good test-retest reliability (Hansson et al., 2009). The PHQ-9 was translated into Norwegian by Pfizer. To our knowledge no study has specifically examined the psychometric properties of PHQ-9. Even so, several studies have used the Norwegian PHQ-9 with adequate results (Solem et al., 2015; Smith et al., 2017). This also applies for the GAD-7. In the current sample, the PHQ-9 showed adequate psychometric properties with a Cronbach's alpha of 0.85.

#### Generalized anxiety disorder, 7-items (GAD-7; Spitzer et al., 2006)

The GAD-7 is a brief 7-item self-report tool developed on criteria for diagnosing generalized anxiety disorder in DSM-IV. The patient scores the frequency of symptoms from 0 (“Not at all”) to three (“Nearly every day”). Total score ranges from 0 to 21. Evidence suggests good psychometric properties (Spitzer et al., 2006; Swinson, 2006). The GAD-7 was translated into Norwegian by Pfizer. As with PHQ-9, no study has been conducted on the psychometric properties of GAD-7, but studies have also used GAD-7 with adequate results (Solem et al., 2015; Smith et al., 2017). In the current sample, the GAD-7 had a Cronbach's alpha of 0.88.

### Data analysis

Our data analytic approach for assessing the psychometric properties of the DOCS-SF was as follows: First, we compared the OCD and comparison group on the DOCS-SF and other study measures. Independent samples *t*-tests were conducted to compare the groups along the total scores in order to detect the ability of the instruments to discriminate between the OCD and the comparison group. Evidence for validity was then gathered using (a) Pearson correlation coefficients between scores on the DOCS-SF and other measures of OCD (convergent validity; e.g., the OCI-R), and (b) correlations between DOCS-SF and measures of other constructs (discriminant validity; i.e., PHQ-9 and GAD-7). Evidence of differences between correlations were assessed using Fisher r-to-z transformation. Treatment sensitivity was assessed by paired sample *t*-tests of pre- and post-treatment values for a sub-sample of the OCD group. Finally, ROC analysis was used to assess the diagnostic accuracy of the DOCS-SF. The ROC is a graph of the association between sensitivity (the degree to which the instrument positively identifies participants with an actual diagnosis) and 1-specificity (the degree to which the instrument correctly identifies participants without a diagnosis as “negative”), and the area under the ROC curve (AUC) represents how well the instrument performs as a tool discriminating between clinical and a comparison group. An AUC-value of 0.5 indicates that the instrument does not perform better than chance when distinguishing OCD patients from the comparison group. An optimal cut-off point when choosing between sensitivity and specificity can be achieved by calculating the Youden Index (also known as Youden's J), as suggested by Perkins and Schisterman (2006). All analyses were conducted using IBM SPSS Statistics 24.

## Results

### Group comparisons

Table 2 displays mean scores on the DOCS-SF, Y-BOCS, OCI-R, and PHQ-9 for the OCD and comparison group. Between-group *t*-tests (also shown in Table 2) revealed that the OCD sample had significantly higher scores on all measures, including the DOCS-SF, when compared to the comparison group. The far right column of the table presents effect sizes (Cohen's *d*) for the group comparisons. Using (Cohen, 1988) criteria for interpretation, large effects were observed on all study measures. Y-BOCS and DOCS-SF yielded larger effect sizes compared to the OCI-R, PHQ-9, and GAD-7.

**Table 2:** Mean scores on study measures for OCD and comparison group.

**Measure**	**OCD (*N* = 244)**			**Comparison group (*N* = 211)**			***t***	***d***
	***M***	**(range)**	***SD***	***M***	**(range)**	***SD***		
DOCS-SF	26.60	12–40	6.14	4.58	0–31	6.42	35.71^*^	3.51
Y-BOCS	25.69	16–38	4.77	5.31	0–27	6.05	36.17^*^	3.77
OCI-R	27.55	5–68	12.34	8.35	0–43	7.80	17.26^*^	1.91
PHQ-9	12.72	1–26	5.66	5.67	0–27	5.37	11.53^*^	1.28
GAD-7	12.28	0–21	5.13	4.94	0–21	4.59	13.72^*^	1.51

* *p < 0.0001 (two-tailed), OCD > Comparison group*.

*OCI-R, Obsessive-Compulsive Inventory-Revised; GAD-7, Generalized Anxiety Disorder; PHQ-9, Patient Health Questionnaire; Y-BOCS, Yale-Brown Obsessive-Compulsive Scale; DOCS-SF, Dimensional Obsessive Compulsive Scale Short-Form*.

Table 3 shows the frequency of endorsement of different types of symptom dimensions on the DOCS-SF checklist, as well as chi-square tests of the differences. Relative to the non-clinical group, the OCD group evidenced a significantly greater frequency of all OCD symptom dimensions (as assessed by the DOCS-SF checklist) except for symmetry and “other” symptoms.

**Table 3:** Percent of participants endorsing different symptom dimensions on the DOCS-SF checklist.

	**OCD group**	**Comparison group**	
**DOCS-SF Dimensions**	**Percent (%)**	**Percent (%)**	***X*^2^**
Contamination	38.7	10.4	45.11^*^
Responsibility	34.8	10.4	35.45^*^
Unacceptable thoughts	24.5	10.0	15.50^*^
Symmetry/ordering	29.9	24.6	1.45
Other	26.5	25.5	0.05

* *p < 0.0001 (two-tailed), OCD > Comparison group. At the checklist patients can endorse more than one symptom. Consequently the total percentage exceeds 100*.

### Reliability

A summary of the Cronbach's alpha values and inter-item correlations is displayed in Table 4. As can be seen, these values provide evidence of good to excellent reliability of the DOCS-SF in both samples, and at post-treatment and follow-up among the treated OCD patients.

**Table 4:** Reliability estimates for DOCS-SF.

	***n***	**α**
Total study sample	415	0.94
Comparison group	211	0.91
**OCD SAMPLE**		
Before treatment	204	0.76
After treatment^a^	102	0.90
3 months F-UP	67	0.95
6 months F-UP	56	0.94

*F-UP, Follow-up*.

a *For the group setting, after treatment assessment was conducted 1 week after the 4 days of intensive treatment, and for the individual therapy this is 1 week after the last session of their therapy (i.e., before the 3 months follow-up session). 3 and 6 months follow-up follow the same time course*.

### Convergent and discriminant validity evidence

Table 5 presents correlations between scores on the DOCS-SF and the other study measures. As can be seen, in the comparison group, and to some extent in the sample as a whole, DOCS-SF scores were more strongly correlated with other measures of OCD than with measures of other constructs. In the OCD group, however, the DOCS-SF showed moderate to strong correlations with measures of OCD as well as with measures of anxiety and depression. Formal tests of differences between correlations (Fisher r-to-z transformation) were also conducted, and supported these findings. The fisher r-to-z transformations can be found in Appendix (Table B1, Supplementary Material). In the OCD sample none of the relations reached significance for differences. In the total sample the relations between DOCS-SF and Y-BOCS was stronger than any other relations. While DOCS-SF and OCI-R was stronger than DOCS-SF and PHQ-9, but not DOCS-SF and GAD-7.

**Table 5:** Correlations between scores on the DOCS-SF and measures of OCD symptoms and other constructs.

**Measure**	**Group**		
	**Total sample**	**OCD patients**	**Comparison group**
Y-BOCS	0.92	0.50	0.79
OCI-R	0.79	0.45	0.71
PHQ-9	0.71	0.55	0.61
GAD-7	0.76	0.55	0.64

*All correlations significant at p < 0.01 (two-tailed)*.

*OCI-R, Obsessive-Compulsive Inventory-Revised; GAD-7, Generalized Anxiety Disorder; PHQ-9, Patient Health Questionnaire; Y-BOCS, Yale-Brown Obsessive-Compulsive Scale; DOCS-SF, Dimensional Obsessive Compulsive Scale Short-Form*.

### Treatment sensitivity

A sub-sample of the OCD patients (*n* = 88) completed treatment as described in Participants and Procedures. All patients completed symptom measures at 1 week following treatment (post-treatment), and an independent rater conducted the Y-BOCS interview over the telephone. Table 6 summarizes the treatment results. The DOCS-SF was sensitive to the effects of treatment as evidenced by the large effect sizes that were comparable to those observed on the other measures of OCD symptoms (i.e., the Y-BOCS and OCI-R).

**Table 6:** Comparison of pre- and post-treatment scores for patients with obsessive-compulsive disorder who completed exposure and response prevention treatment (*n* = 88).

**Measure**	**Pre-treatment**		**Post-treatment**		***d*^†^**
	***M***	***SD***	***M***	***SD***	
Y-BOCS	25.53	4.65	10.07	4.81	2.44^*^
DOCS-SF	26.31	5.77	12.43	8.25	1.67^*^
OCI-R	25.81	11.66	10.68	8.45	1.47^*^

* *p < 0.0001 (two-tailed), Pre-treatment > Post-treatment*.

† *Cohen's d was calculated using average of pre and post-treatment standard deviation, and corrected for dependence between means. OCI-R, Obsessive-Compulsive Inventory-Revised; G; Y-BOCS, Yale-Brown Obsessive-Compulsive Scale; DOCS-SF, Dimensional Obsessive Compulsive Scale Short-Form*.

### Receiving operating characteristics

**Figure 1:**
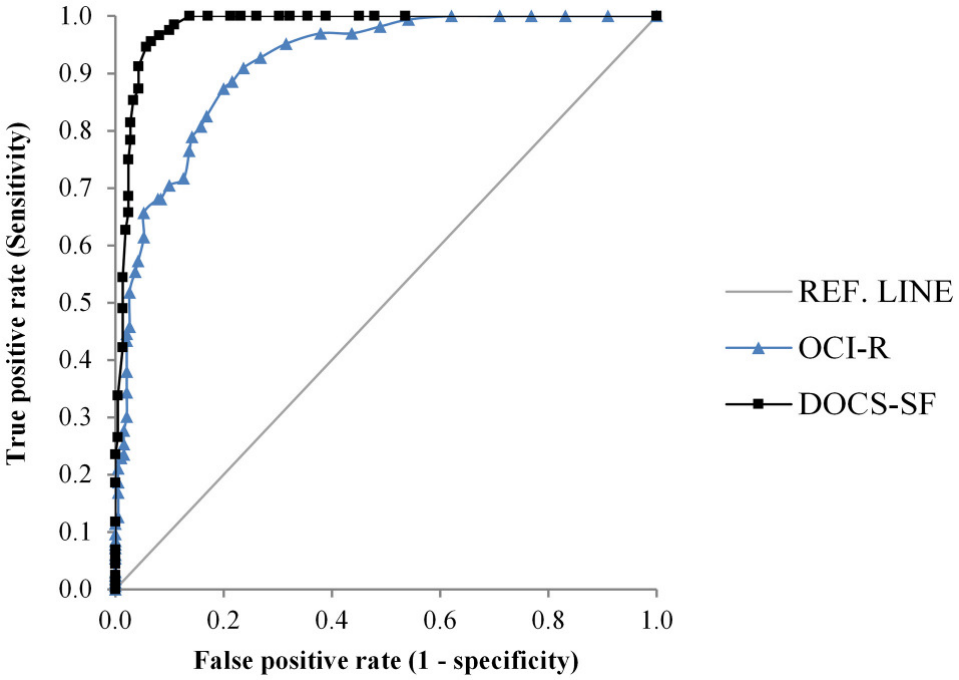
Receiver operating characteristic curves for DOCS-SF and OCI-R.

We computed the AUC for the DOCS-SF and for the OCI-R (the only other self-report measure of OCD symptoms in the present study) as a comparison as shown in Figure 1. We found that the AUC for the DOCS-SF was 0.98 [95% CI 0.97–0.99], suggesting that the test discriminated extremely well between patients diagnosed with OCD and the comparison group. We compared the AUC between the DOCS-SF and that of the OCI-R, which was 0.92 (95% CI 0.89–0.95), and found a significant difference (*p* < 0.0001). Thus, the DOCS-SF performed significantly better relative to the OCI-R.

Based on the Youden Index the analysis suggested a cut-off score of 16. This score had a sensitivity of 96% [95% CI 92–98] and a specificity of 94% [95% CI 89–96], indicating a good ability to correctly identify those in the OCD group from those in the comparison group.

## Discussion

The current study examined the psychometric properties of a Norwegian short version of the original DOCS. Based on a scale that assesses the severity of these four best-supported symptom dimensions, the main objective behind the design of the DOCS-SF was to create a measure that could provide a brief (5-item) measure of OCD symptom severity to aid carrying out rapid screening of OCD or making repeated assessments. Overall, our findings suggest a psychometrically sound and stable instrument.

First, the DOCS-SF was able to distinguish between OCD patients and the comparison group, providing evidence of criterion validity. It is noteworthy that on the DOCS-SF symptom checklist, there were between-group differences in the frequency of endorsement of contamination, responsibility, and unacceptable thoughts symptoms, but no differences between patients and comparison group on the Symmetry/Ordering and Other symptom dimensions. Perhaps this reflects the high prevalence of symmetry and ordering symptoms among non-clinical samples (e.g., Abramowitz et al., 2014). With regards to the “Other” category, it is possible that both groups endorsed this category on the basis of non-OCD symptoms (e.g., depressive or other anxiety symptoms). The lack of examples in the DOCS-SF to guide the endorsement of these categories might explain the relatively weak evidence for discriminative validity as discussed further below.

Despite the fact that DOCS-SF contains only five items, we found adequate support for internal consistency, indicating that the five items measure the same general construct. This was not surprising given evidence that similar parameters of OCD symptom severity were found to be reliable and valid indices of severity on the original DOCS.

Scores on the DOCS-SF also correlated with other self-report (OCI-R and Y-BOCS) and clinician-interview (Y-BOCS) measures of OCD, providing strong evidence of convergent validity for assessing such phenomena. At the same time, scores on the DOCS-SF correlated strongly with both PHQ-9 and GAD-7, with more disparity for the comparison group compared to the OCD sample. Test for the difference of correlations provided further evidence of this. This suggests mixed evidence for discriminant validity, which might be especially lacking among individuals with a clinical diagnosis of OCD. Perhaps the comorbid conditions within members of the OCD group accounted for this, as well as a restriction of range in OCD symptoms at pre-treatment (all participants had moderate to severe OCD symptoms).

The DOCS-SF was sensitive to treatment effects as indicated by a pre-post treatment effect size of 1.67. Furthermore, the analysis of receiver operating characteristics provided strong evidence for the diagnostic ability of DOCS-SF. A suggested cut-off score of 16 of the DOCS-SF provided the best combination of sensitivity and specificity for identifying OCD patients from non-patients. This is the same cut-off score as used for the Y-BOCS. Applying the cut-off score 64% of patients were considered non-clinical post-treatment using the DOCS-SF. The similar score for OCI-R were 63% (Cut-off of 12; Wootton et al., 2015). Using empirically derived criteria for Y-BOCS 76% had “mild symptoms” (0–13; Storch et al., 2015). In summary, these findings should support the use of DOCS-SF as a tool for measuring changes between sessions, and GPs could use it as a quick screening tool for OCD symptoms before they refer patients to specialist health care.

The present study has a number of limitations that should be considered alongside our findings. First, we used convenience sampling to recruit the comparison group, and the samples were recruited using different methods, which could introduce a selection bias. While there were no differences in sex and age between the two samples, the comparison group is not necessarily representative of the general population. Still, the mean score for the comparison group on OCI-R is similar to the number found for the comparison group in the Norwegian validation of the questionnaire (*M* = 8.35 ± 7.80 vs. *M* = 10.44 ± 9.18; Solem et al., 2010). Second, the lack of a psychiatric comparison group to test whether our findings for the OCD group were specific to this particular disorder is a limitation. A third shortcoming is the lack of test-retest reliability. Fourth, comparison group participants were not face-to-face assessed for psychological disorders and therefore it cannot be excluded that they satisfied diagnostic criteria. Fifth, the measures used to test convergent and discriminant validity have not yet been properly validated into Norwegian. Finally, the comparison group used the self-report Y-BOCS rather than the Y-BOCS interview, which was used to assess the OCD group.

There are some unanswered questions concerning DOCS-SF. We recommend future studies to use diagnostic interviews for the comparison group population as well, thus strengthening the ROC analysis. More research is needed to explore the possible weak support for discriminant validity of the DOCS-SF in OCD samples as this may be a result of comorbidity and restriction of range. In conclusion, results from the current study should warrant further clinical use and research on this brief self-report tool for OCD symptoms.

## Author contributions

TE was responsible for the data analysis, interpretation, drafting, and revising the work. BH and GK designed the questionnaire and project and was responsible for the data collection. JA created the original DOCS, gave us permission to create the DOCS-SF inspired by DOCS, and contributed to interpretation and gave insightful comments during manuscript revisions. SH participated in the interpretation and revision process of the manuscript. SS participated in designing the project, interpretation and revising the manuscript. All authors gave their final approval of the version to be published.

### Conflict of interest statement

The reviewer GB and handling Editor declared their shared affiliation, and the handling Editor states that the process nevertheless met the standards of a fair and objective review. The other authors declare that the research was conducted in the absence of any commercial or financial relationships that could be construed as a potential conflict of interest.

## Abbreviations

OCD: Obsessive-compulsive disorder
DOCS: Dimensional Obsessive-Compulsive Scale
DOCS-SF: Dimensional Obsessive Compulsive Scale-Short Form
OCI-R: Obsessive-Compulsive Inventory-Revised
Y-BOCS: Yale-Brown Obsessive-Compulsive Scale
GAD-7: Generalized Anxiety Disorder, 7-items
PHQ-9: Patient Health Questionnaire, 9-items
ROC: Receiver Operating Characteristics
CSSRI: Socio-Demographic and Service Receipt Inventory
AUC: Area Under the Curve.
