# Supplementary material for: The Dimensional Obsessive-Compulsive Scale: Development and Validation of a Short Form (DOCS-SF)
Source: Front Psychol. 2017 Sep 5;8:1503. doi: 10.3389/fpsyg.2017.01503 (PMC5591872; doi:10.3389/fpsyg.2017.01503)
Supplement: Supplementary file 1 [file DataSheet1.docx]

# Appendix A

Appendix A1.


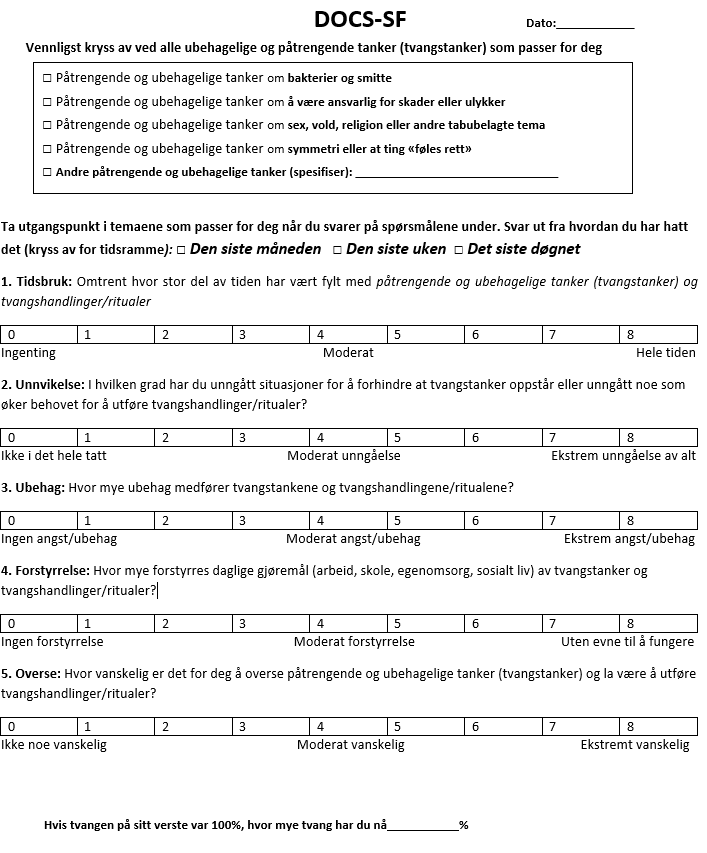


# Appendix B

| Table B1 | | | | | | | |
| --- | --- | --- | --- | --- | --- | --- | --- |
| *Testing significance of difference between two correlation coefficients for DOCS-SF and to measures of OCD symptoms and other constructs using Fisher r to z transformations* | | | | | | | |
| Measure 1^a^ | *r* |  | Measure 2^b^ | *r* |  | *z* | *p^c^* |
| Total sample | | | | |  |  |  |
| Y-BOCS | .92 |  | OCI-R | .79 |  | 7 | < .0001 |
| Y-BOCS | .92 |  | PHQ-9 | .71 |  | 9.28 | < .0001 |
| Y-BOCS | .92 |  | GAD-7 | .76 |  | 7.87 | < .0001 |
| OCI-R | .79 |  | PHQ-9 | .71 |  | 2.40 | .02 |
| OCI-R | .79 |  | GAD-7 | .76 |  | 0.98 | .33 |
| PHQ-9 | .71 |  | GAD-7 | .76 |  | 1.39 | .17 |
|  |  |  |  |  |  |  |  |
| OCD Patients | | | | | | | |
| Y-BOCS | .50 |  | OCI-R | .45 |  | 0.61 | .54 |
| Y-BOCS | .50 |  | PHQ-9 | .55 |  | 0.63 | .53 |
| Y-BOCS | .50 |  | GAD-7 | .55 |  | 0.63 | .53 |
| OCI-R | .45 |  | PHQ-9 | .55 |  | 1.21 | .23 |
| OCI-R | .45 |  | GAD-7 | .55 |  | 1.22 | .22 |
| PHQ-9 | .55 |  | GAD-7 | .55 |  | 0 | 1 |
|  |  |  |  |  |  |  |  |
| Comparison group | | | | | | | |
| Y-BOCS | .79 |  | OCI-R | .71 |  | 1.75 | .08 |
| Y-BOCS | .79 |  | PHQ-9 | .61 |  | 3.43 | < .001 |
| Y-BOCS | .79 |  | GAD-7 | .64 |  | 2.97 | < .01 |
| OCI-R | .71 |  | PHQ-9 | .61 |  | 1.71 | .09 |
| OCI-R | .71 |  | GAD-7 | .64 |  | 1.24 | .22 |
| PHQ-9 | .61 |  | GAD-7 | .64 |  | 0.47 | .64 |
| *Note*. ^a^ This is the first correlation of the Fisher r to z transformation, e.g. between DOCS-SF and Y-BOCS. ^b^ This is the second correlation used, e.g. DOCS-SF and OCI-R. ^c^This is the p-value for the difference between the first and the second correlation. | | | | | | | |
